# Nano Size Effects of TiO_2_ Nanotube Array on the Glioma Cells Behavior

**DOI:** 10.3390/ijms14010244

**Published:** 2012-12-21

**Authors:** He Yang, Xiaofei Qin, Ang Tian, Dongyong Zhang, Xiangxin Xue, Anhua Wu

**Affiliations:** 1 Liaoning Provincial Universities Key Laboratory of Boron Resource Ecological Utilization Technology and Boron Materials, Northeastern University, No. 11, Lane 3, Wenhua Road, Heping District, Shenyang 110819, China; E-Mails: Yangh@smm.neu.edu.cn (H.Y.); Xuexx@mail.neu.edu.cn (X.X.); 2 Departments of Neurosurgery, The First Affiliated Hospital of China Medical University, 155 Nanjingbei Street, Heping District, Shenyang 110001, China; E-Mails: claudeqin@msn.com (X.Q.); dongyong0622@hotmail.com (D.Z.)

**Keywords:** nanotube, glioma cells, proliferation, inflammatory mediator’s gene, signal channel

## Abstract

In order to investigate the interplay between the cells and TiO_2_ nanotube array, and to explore the ability of cells to sense the size change in nano-environment, we reported on the behavior of glioma C6 cells on nanotube array coatings in terms of proliferation and apoptosis. The behavior of glioma C6 cells was obviously size-dependent on the coatings; the caliber with 15 nm diameter provided effective spacing to improve the cells proliferation and enhanced the cellular activities. C6 cells’ biological behaviors showed many similar tendencies to many phorocytes; the matching degree of geometry between nanotube and integrin defined that a spacing of 15 nm was optimal for inducing signals to nucleus, which results in achieving maximum activity of glioma cells. In addition, the immune behavior of cells was studied, a variety of inflammatory mediator’s gene expression levels were controlled by the nanoscale dimension, the expressions of IL-6 and IL-10 were higher on 30 nm than on 15 nm nanotube.

## 1. Introduction

A great number of researches have established that most phorocytes possess intensely strong recognition on the change of shape on nanometer scale of embedding materials [1,2]; such recognition is essential to cells migration, multiplication and growth [3,4]. In contrast to polystyrene or smooth TiO_2_ coating surface, TiO_2_ nanotube (TNT) array coating would further enhance cell’s activity on material surface [5–7], especially the mesenchymal stem cells [8], hemopoietic stem cells [9], smooth muscle cells [10], endothelial cells [11], osteoblasts [12] and other phorocytes. Research indicates that phorocytes are obviously dependent on the pipe diameter of TNT nanometer tube; however, there has been controversy surrounding this principle [11]. For instance, various researches have indicated that optimal pipe diameters of strengthened osteoblast are 15 nm [8], 50 nm [13], 70 nm [14], 80 nm [15]. In addition, research on mesenchyme stem cells has concluded similarly controversial results [2,16,17]. Popat and other researchers [16] argue that 80 nm pipe diameter would maximize the multiplication and absorption of bone marrow stromal cells and the activity of alkaline phosphatase. Further, Oh *et al.* [17] have demonstrated that although a 30 nm pipe diameter may enhance the multiplication of mesenchyme stem cells to some extent, it cannot effectively induce the cells’ differentiation while 70 nm pipe diameter could induce and initiate stem cells’ differentiation into osteoblasts. In contrast, Park *et al.* [2] have indicated that when the diameter of the nanotube is around 15 nm, the activity of all phorocytes would be greatly enhanced; while when the diameter is over 50 nm, cells would exhibit apoptosis.

Up to now, there have been reports on how to solve the problems mentioned above such as the preparation method of the material, of sterilization and on TNT wettability’s influence upon cell action. However, such reports raise further controversy apart from establishing the cell’s dependence on nanotube optimum diameter. For instance, different approaches of sterilization generate different TNT surface wettability and surface energy. Some researchers argue that such differences influence the cell’s multiplication and adsorption [11]; while other researchers argue that the cell’s action depends solely on the TNT tube diameter’s size [8]. Although the interaction between TNT and cells needs more exploration, on the basis of past researches we can now assume that TNT’s influence on cell biological actions has obvious cell selectivity and that the differences among cells might enable uncovering the causes as to why there have been differences among TNT surface cells’ recognition capability. Among all the primary brain tumors, glioma is a kind of tumor whose morbidity is highest and is the most difficult to diagnose. Research on epidemiology has shown that although morbidity of glioma varies immensely, it accounts for almost half of all the primary cerebral tumors and this tendency appears to be increasing [18]. Cerebral glioma C6 cell has been chosen as a subject to explore whether the cells’ recognition on the nanometer scale micro-environmental change is an inter-species universal behavior, in the hope of finding this kind of cell’s unique biological trait.

## 2. Results and Discussion

**Figure 1:**
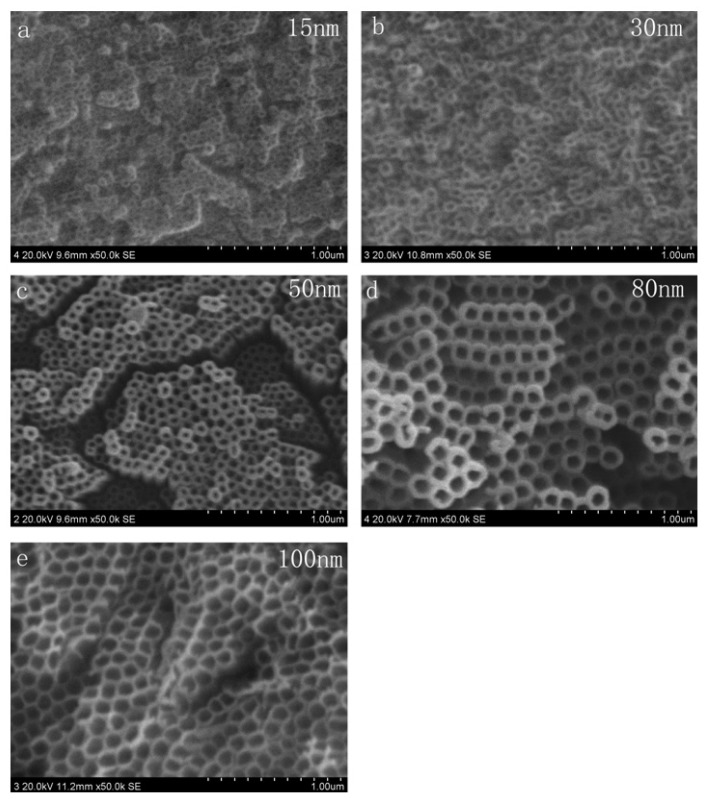
SEM micrographics of morphology of TiO_2_ nanotube (TNT) coatings prepared by different oxidation voltages: (**a**) 20 V; (**b**) 30 V; (**c**) 40 V; (**d**) 50 V; (**e**) 60 V.

**Figure 2:**
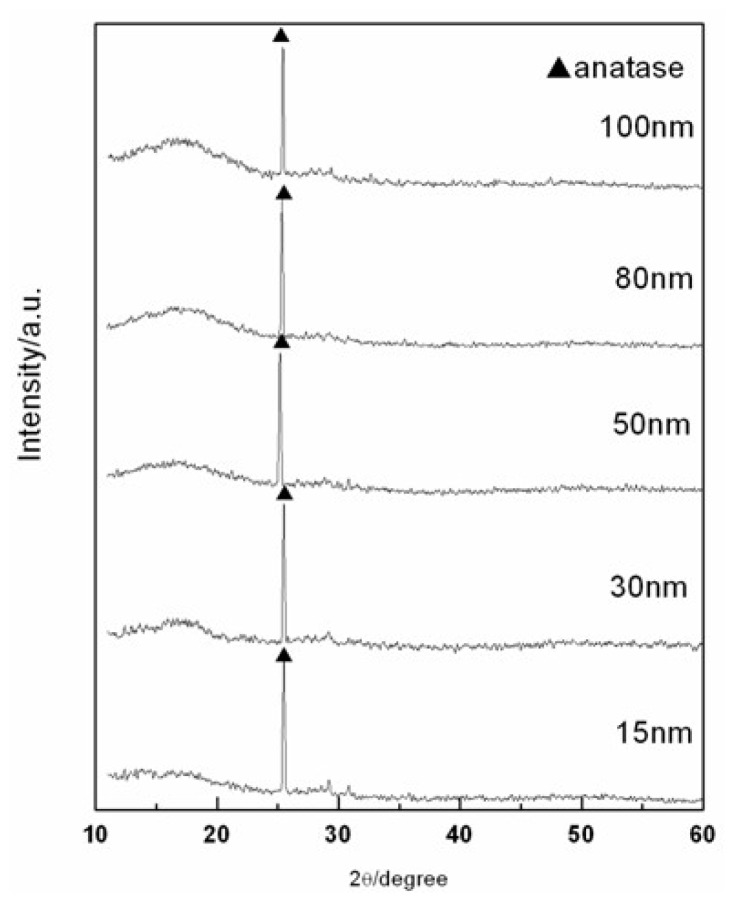
XRD analysis of TNT. All samples are found to be anatase in an amorphous background.

**Figure 3:**
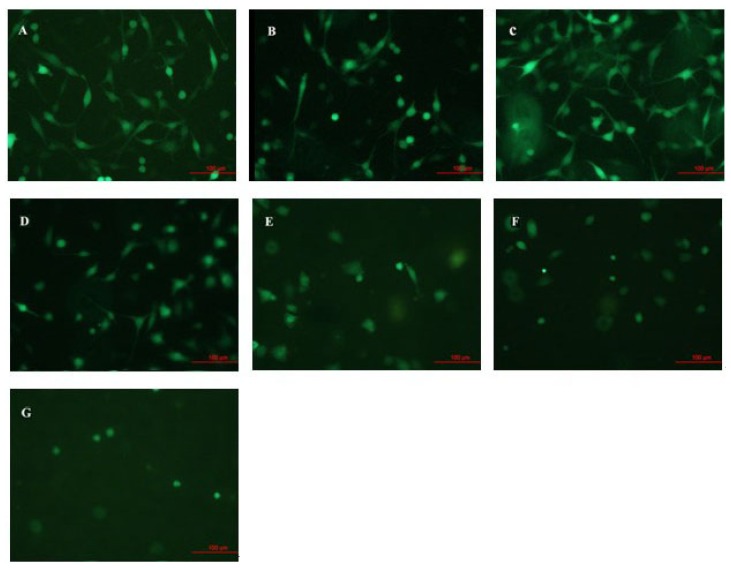
The growing states of C6 glioma cell lines under the fluorescence microscope after being cultured 24 h on the surface of TiO_2_ nanotube array of different shapes (20 times magnification). (**A**) Plastic sheet prepared from polylysine; (**B**) Ti; (**C**) 15 nm-TNT; (**D**) 30 nm-TNT; (**E**) 50 nm-TNT; (**F**) 80 nm-TNT; (**G**) 100 nm-TNT.

The variation of C6 cells action after being cultivated for 24 h on the surfaces of TNT array with different diameters has been observed, as Figure 1 shows. The TNT array films were fabricated by the anodized method. The diameter of nanotube increased with the increase of voltage, which caused by the electrochemical anodization speed increasing [19]. The results of XRD (as Figure 2 illustrates) indicated that all the TNT samples typically show the same structure which has an amorphous phase with some anatase phases. After that, cells have been fixed and applied with immunofluorescence staining. In our experiment, we set Ti and poly-lysine as the control group. Figure 3 infers that the tube diameter in TNT membranes determines cell density. The number of cells on the poly-lysine plastic surface, Ti surface and sample with an average of ~15 nm inner diameter (abbreviated 15 nm-TNT) show no apparent difference; however, 30 nm nanotube diameter is the separation on cell density change: the bigger the tube diameter, the less the number of cells on the sample surface. In addition, we have found that the C6 cells cultivated on 15 nm-TNT, Ti and poly-lysine surface have shown a typical cell migration trend. They are able to migrate on the surface cells, forming lamellipodia and wide thick filaments of locomotion. When the tube diameter is over 30 nm, cell absorption and migration show a trend to decline: the bigger the diameter is, the less the C6 cells which possess filaments of locomotion can be observed. When the cells were seeded on a nanotube surface with 80–100 nm, the adhesion of cells was the lowest.

**Figure 4:**
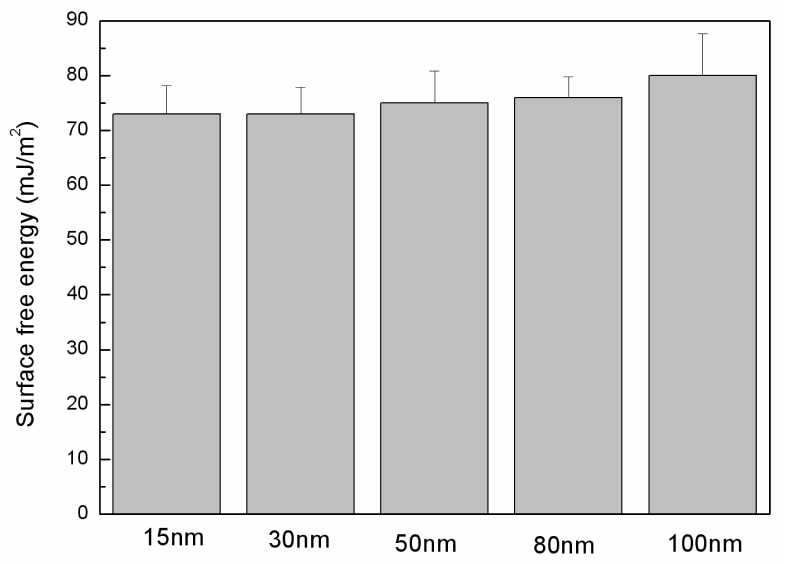
Values of surface free energy of the TNT samples after being sterilized in ethanol (mJ/m^2^).

On different tube diameters of TiO_2_ nanotube array surfaces, C6 cells’ biological behaviors show many similar tendencies to phorocytes [2,8]. A diameter of around 15 nm produces the most cells and best cell state on TiO_2_ nanotube array surface; however, when the tube diameter becomes bigger, there is an apparent decline in both cell population and state. Bauer and others’ research [8,9] have indicated that in comparison to 50 nm-TNT surface, substantial focal contact has been detected in marrow mesenchyme stem cells which grow on 15 nm-TNT surface. The development of focal contact is due to significant paxillin secreted in cells. Paxillin is a kind of focal adhesion protein, which attach integrin to the actin fiber network, enabling integrin protein access into the focal contact complex, activating mesenchymal cell signal transfer into nucleus and cytoskeleton [20,21]. This process can control cell adsorption, form, multiplication and migration. 15 nm-TNT array samples can induce C6 cell producing pile protein, promoting integrin protein integration into complex. In addition, the values of the surface free energy were calculated to explore whether this factor would affect the cell’s behavior. The surface free energy is one of the main factors that would influence the interaction between the cell and anodized titanium [22]. The surface free energies of samples are displayed in Figure 4. The highest value (about 78 mJ/m^2^) was in a sample of 100 nm diameter; next came 50 nm and 80 nm nanotube samples, with about 75 mJ/m^2^; the 15 nm and 30 nm samples tend to have the lowest values, with about 72 mJ/m^2^. The difference of values between all the samples is not obvious, as the ethanol immersion would lead to the hydrophilicity and alter the surface characteristic [11], the sterilization by ethanol results in a similar surface free energy among all the TNT surfaces. Thus it can be inferred that in a TiO_2_ nanotube array constructed culture system, C6 biological behavior is sensitive to the change in nanotube diameter, exhibiting an apparent size dependent effect.

**Figure 5:**
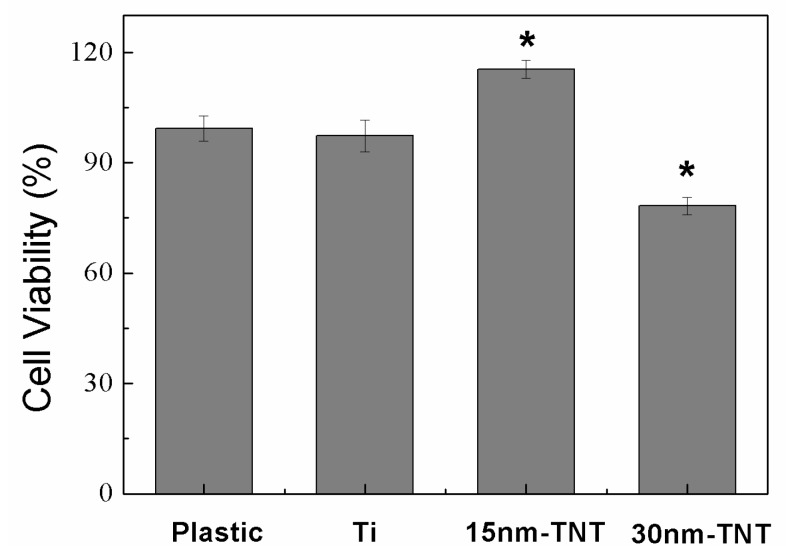
MTT test on the influence of livability of C6 cell on TiO_2_ nanotube array (*p < 0.05).

The MTT experiment set cell culture board and Ti as control group, and set 15 nm-TNT and 30 nm-TNT as culture cell baseboard, with a control after 24 hours’ culture. From Figure 5 it can be inferred that the survival rates of Ti and cells on ordinary cell culture board show no apparent statistical difference (*p* < 0.05), and that cells on 15 nm tube diameter sample surface shows the highest survival rate, which differs from the control group (*p* < 0.05); and that when the tube diameter is 30 nm, cell survival rate is lower than the ordinary culture board group (*p* < 0.05). MTT has further proved that 15 nm is the most suitable tube diameter for C6 cell growth; compared with poly-lysine culture dish and Ti, 30 nm tube diameter inhibits cell multiplication to some extent. Previous studies [8,9] indicate that for osteoblast and mesenchyme stem cells cultured on TiO_2_ nanotube array surface, when nanotube diameter is over 70 nm, cell multiplication and differentiation have been apparently inhibited; while when nanotube diameter is between 15 and 30 nm, phorocytes have shown most active proliferation. Such a result is inconsistent with our experiment results. Although glioma cells have the same apparent size dependence as tissue cells, different cell clones determine different cell behavior size limitations. Nano space determines cell activity; the simulation model [2] has shown that different nanotube diameters determine different crosswise spaces of contact. A 15 nm space seems to be optimal for integrating protein into contact, thus promoting actin fiber assembly and cell nucleus signal transfer. When the nanotube diameter is over the critical dimensions, contact development and cell signal transfer will be disabled, thus causing cell apoptosis.

**Figure 6:**
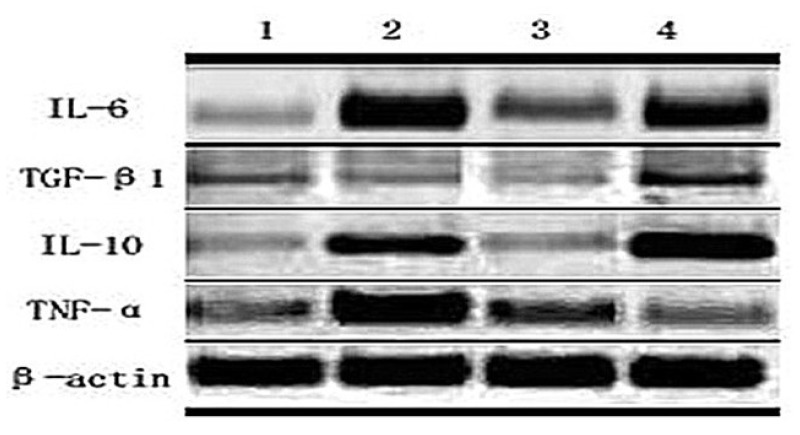
Expression of mRNA in the C6 cell cultured 24 h on the TiO_2_ nanotube array with different diameters: (**1**) 15 nm-TNT; (**2**) 30 nm-TNT; (**3**) Ti; (**4**) control group: cell culture plate of plastic prepared from polylysine.

With deeper research into immunology, it can be concluded from the constant expression and secretion of cultured glioma cell factor that the occurrence and development of brain glioma relates to the abnormal expression and secretion of some cytokine genes. Cell factor expression and generation involve not only immune cell multiplication and differentiation, but also several internal cells growth, multiplication and metabolism adjustment. Glioma cells express many kinds of cell factors during the course of occurrence, development, invasion and transfer which can either enhance or inhibit immunocyte growth [23,24]. Auxiliary T cell can secrete several kinds of cell factors. Th1 cell mainly secretes interleukin, interferon, tumor necrosis factor-α and TNF-β, taking part in cell immune and delayed-type hypersensitive inflammation; Th2 cell mainly secretes IL-4, IL-5, IL-6, IL-9, IL-10, IL-13 and so on, assisting B cell differentiating into antibody secretory cells, taking part in humoral immune response. In this paper, we take TiO_2_ nanotube array as cell culture scaffold, and rat C6 cell TNF-α, TGF-β1, IL-10 and IL-6 expression has been examined by Reverse Transcription-Polymerase Chain Reaction, as Figure 6 showed, in the hope of signifying the relationship between topography of TNT and immune behavior. According to the result of RT-PCR, C6 glioma cells, after being cultivated for 24 h on different sample surfaces, have shown corresponding differences in various inflammatory mediators’ expression levels. Different inflammatory mediators’ genes have different trends in expression level: IL-10 and TGF-β1 share the same trend; and TNT can inhibit both the IL-10 and TGF-β1 mRNA expression. For IL-6 mRNA expression, the 15 nm-TNT group shows an apparent decline while 30 nm-TNT shows an obvious increase. L6 and IL10 attribute to Th2 type inflammatory factor, their expression level is relative to the tube diameter. IL-6 and IL-10 on poly-lysine plastic cardinal surface have shown higher expression level than on 15 nm and pure Ti surface. 30 nm-TNT may also enhance TNF-α mRNA expression while 15 nm-TNT group shows no significant difference from control group. As the tube diameter increases, TGF-β1 shows no difference in expression level while pure titanium surface shows a lower expression level and the plastic surface shows a higher expression level than any other samples.

It has been found in recent years that tumor tissue mostly secretes Th2 Type cell factor, which imposes both inhibition and stimulation upon tumor activity. It has also been found that an organism in Th2 cell factor predominant conditions is a mechanism of tumor immune escape, which is hostile for organism anti-tumor immune response in the form of cell immunity [25]. As the RT-PCR results show, 15 nm-TNT could inhibit Th2 cell factor; meanwhile, it has no influence on Th1 cell factor. Combined with MTT results, we could infer that the Th2 cell factor seemingly prefers inhibiting the activity of the cells rather than stimulating cells in a TNT constructed cultured system. Additionally, it is reasonable to suppose that the topography of the nanotube array could affect the Th2 cell factor secretion and influence the behavior of glioma cells. Of course, the above hypotheses will be proven in future related experiments.

## 3. Experimental Section

### 3.1. TNT Formation with Different Diameters

99.5% commercial titanium foil (Sigma-Aldrich) of 500 μm thick was cut into discs with 10 mm in diameter and were washed with ethanol and acetone for about 10 min by ultrasonication to remove the contamination. Then the foils were thoroughly rinsed with distilled water and dried under the nitrogen atmosphere prior to anodization. Two-electrode configuration was used to anodize the titanium foils in a thermostated bath of 75 cm^3^ volume, and platinum was served as a counter electrode, the distance between working electrode and counter electrode was 4 cm. For anodizing, a controlled DC power source (SKD-200 V, Shanghai SanKe, Shanghai, China) supplied the required constant potential. Anodization was carried out in an electrolyte solution containing ethylene glycol, hydrofluoric acid, and 0.5 wt % NH4F with varied voltages as noted below: 20 V, 30 V, 40 V, 50 V and 60 V. HF concentration and temperature at constant values of 0.75 vol.% and 20 °C were used. To decrease the defect of the titanium surface, the foils were pre-anodized [19] at 60 V for 2 h in a 0.5 wt % NH4F ethylene glycol solution followed by ultrasonication in 1 M HCl aqueous solution to remove the films. The samples after anodized by the two-step anodization procedure were washed by deionized water and then dried in air. The samples were observed under a FESEM (Hitachi) to record the surface morphology. The nanotube coating was scraped from the Titanium substrate, and the phase information of the samples was analyzed using X-ray diffraction (PANalytical, X’Pert Pro x-ray diffractometer) method using Cu K_α_ radiation. Ultra-pure water was employed in the contact angle measurements; the contact angles were measured with the equipment consisted by a camera and a microscope. The free energy of each surface was calculated according to the reference [22].

### 3.2. Cell Culture

For cell proliferation assay, TNT samples with different size of caliber which were sterilized by ethanol immersion were put in culture plate. C6 glioma cells of logarithmic phase were seeded in 6-well plates with TiO_2_ particles of different sizes, at a density of 5 × 10^5^ cells/well for 24 h, and then MTT (Sigma-Aldrich, Inc., New York, NY, USA, 200 μL, 5 mg/mL) was added. The medium was not changed during the incubation. After 4 h incubation, the medium was removed by aspiration and formazan crystals were dissolved in 1.5 mL of solubilizing reagent DMSO (Sigma-Aldrich, Inc., New York, NY, USA). After shaking for 10 min, the solution in each well was transferred into 96-well plates (300 μL/well). Absorbance was measured at 490 nm using an enzyme immunoassay instrument. All experiments were repeated three times and cell viability was then determined by the following equation:

(1) cell viability (%)=(Abs test cells/Abs control cells)×100

### 3.3. Immunocytochemistry Staining of C6 Glioma Cells

Briefly, C6 glioma cells were re-seeded in 6-well plates with TiO_2_ of different diameters at a density of 5 × 105 cells/well for 24 h. Then all the cells were fixed using 4% paraformaldehyde at room temperature for 30 min, and washed three times with PBS. Next, the cells were permeabilized with 0.3% Triton-X-100 (Sinopharm Corp., Shanghai, China) for 30 min, washed with PBS, and incubated in 5% bovine serum albumin (Zhongshan Goldenbridge Biotechnology Corp., Beijing, China) for 20 min. After removal of the blocking solution, the cells were incubated at 4 °C overnight in 1:200 rabbit anti-rat GFAP (glial fibrillary acidic protein, Chemicon International, Temecula, CA, USA). After washed by PBS, the cells were incubated at 37 °C for 2 h with 1:100 FITC-conjugatedgoat anti-rabbit IgG (Sigma-Aldrich, Inc., New York, NY, USA) and examined under a fluorescence microscope. In control samples, primary antibody was replaced by isotypeIgG.

### 3.4. Semiquantitative Detection of mRNA with Reverse Transcription Polymerase Chain Reaction (RT-PCR)

All samples of cells which were cultured on TNT for 48 h were placed on ice, from which RNA was extracted using TRIzol reagent (Invitrogen, Carlsbad, CA, USA). RNA (500 ng) was reverse transcribed into cDNA. The primer sequences of TNF-α were as follows: 5′-CCA CGC TCT TCT GTC TAC TG-3′ for the forward primer and 5′-GCT ACG GGC TTG TCA CTC-3′ for the reverse primer, giving a 145 bp amplified fragment. The primer sequences of TGF-β1 were as follows: 5′-CCG CAA CAA CGC AAT CTA-3′ for the forward primer and 5′-TGA GGA GCA GGA AGG GTC-3′ for the reverse primer, giving a 437 bp amplified fragment. The primer sequences of IL-10 were as follows: 5′-GCT ATG TTG CCT GCT CTT-3′ for the forward primer and 5′-ATG CTC CTT GAT TTC TGG-3′ for the reverse primer, giving a 307 bp amplified fragment. The primer sequences of IL-6 were as follows: 5′-CCT TCT TGG GAC TGA TGT-3′ for the forward primer and 5′-CTC TGG CTT TGT CTT TCT-3′ for the reverse primer, giving a 384 bp amplified fragment. The primer sequences of β-actin were as follows: 5′-GAG ACC TTC AAC ACC CCA GC-3′ for the forward primer and 5′-CCA CAG GAT TCC ATA CCC AA-3′ for the reverse primer, giving a 531 bp amplified fragment. Condition for reverse transcriptase PCR was as followed: [95 °C for 5 min, (95 °C for 30 s, 55–60 °C for 30 s, and 72 °C for 30 s) × 30 cycles, 72 °C for 10 min]. PCR products were visualized by electrophoresis on an ethidium bromide-stained (0.5 μg/mL) 1.6% agarose gel and photographed under ultraviolet light.

## 4. Conclusions

In the present work we demonstrate that the biological behavior of glioma cells on nanotube array layers are in line with phorocytes on TiO_2_ nanotubes. The results of our research indicated that glioma cells adhesion, proliferation and activity are several-fold higher on 15 nm than on nanotubes with larger diameters; also the state of cells exhibited a declining trend with an increase in pipe diameter. Additionally, after being cultured on the TiO_2_ nanotube array, the expression of each immune factor differed. We hypothesize that the topography of TNT could influence the Th2 type cell factors, which potentially inhibits the activity of glioma cells to some extent.

## References

[1] Peng L.L., Eltgroth M.L., LaTempa T.J., Grimes C.A., Desai T.A. (2009). The effect of TiO_2_ nanotubes on endothelial function and smooth muscle proliferation. Biomaterials.

[2] Park J., Bauer S., von der Mark K., Schmuki P. (2007). Nanosize and vitality: TiO_2_ nanotube diameter directs cell fate. Nano Lett.

[3] Brammer K.S., Oh S., Gallagher J.O., Jin S.H. (2008). Enhanced cellular mobility guided by TiO_2_ nanotube surfaces. Nano Lett.

[4] Popat K.C., Leoni L., Grimes C.A., Desai T.A. (2007). Influence of engineered titania nanotubular surfaces on bone cells. Biomaterials.

[5] Lai M., Cai K., Hu Y., Yang X., Liu Q. (2012). Regulation of the behaviors of mesenchymal stem cells by surface nanostructured titanium. Colloids Surf. B.

[6] Oh S., Brammer K.S., Moon K., Bae J.M., Jin S. (2011). Influence of sterilization methods on cell behavior and functionality of osteoblasts cultured on TiO_2_ nanotubes. Mater. Sci. Eng. C.

[7] Khang D., Park G.E., Webster T.J.J. (2008). Enhanced chondrocyte densities on carbon nanotube composites: The combined role of nanosurface roughness and electrical stimulation. Biomed. Mater. Res. A.

[8] Bauer S., Park J., Faltenbacher J., Berger S., von der Mark K., Schmuki P. (2009). Size selective behavior of mesenchymal stem cells on ZrO_2_ and TiO_2_ nanotube arrays. Integr. Biol.

[9] Park J., Bauer S., Schlegel K.A., Neukam F.W., von der Mark K., Schmuki P. (2009). TiO_2_ Nanotube surfaces: 15 nm-an optimal length scale of surface topography for cell adhesion and differentiation. Small.

[10] Park J., Bauer S., Schmuki P., von der Mark K. (2009). Narrow window in nanoscale dependent activation of endothelial cell growth and differentiation on TiO_2_ nanotube surfaces. Nano Lett.

[11] Zhao L.Z., Mei S.L., Wang W., Chu P.K., Wu Z.F., Zhang Y.M. (2010). The role of sterilization in the cytocompatibility of titania nanotubes. Biomaterials.

[12] Yu W.Q., Zhang Y.L., Xu L., Sun S.J., Jiang X.Q., Zhang F.Q. (2012). Microarray-based bioinformatics analysis of osteoblasts on TiO_2_ nanotube layers. Colloids Surf. B.

[13] Das K., Bose S., Bandyopadhyay A. (2009). TiO_2_ nanotubes on Ti: Influence of nanoscale morphology on bone cell—Materials interaction. J. Biomed. Mater. Res. A.

[14] Balasundaram G., Yao C., Webster T.J. (2008). TiO_2_ nanotubes functionalized with regions of bone morphogenetic protein-2 increases osteoblast adhesion. J. Biomed. Mater. Res. A.

[15] Popat K.C., Eltgroth M., Latempa T.J., Grimes C.A., Desai T.A. (2007). Decreased *Staphylococcus* epidermis adhesion and increased osteoblast functionality on antibiotic-loaded titania nanotubes. Biomaterials.

[16] Popat K.C., Leoni L., Grimes C.A., Desai T.A. (2007). Influence of engineered titania nanotubular surfaces on bone cells. Biomaterials.

[17] Oh S., Brammer K.S., Li Y.S.J., Teng D., Engler A.J., Chien S., Jin S. (2009). Stem cell fate dictated solely by altered nanotube dimension. Proc. Natl. Acad. Sci. USA.

[18] Ohgaki H., Kleihues P. (2005). Epidemiology and etiology of gliomas. Acta Neuropathol.

[19] Wang D.A., Ling Y., Yu B., Zhou F., Liu W.M. (2009). TiO_2_ Nanotubes with tunable morphology, diameter, and length: Synthesis and photo-electrical/catalytic performance. Chem. Mater.

[20] Schlaepfer D.D., Hauck C.R., Sieg D.J. (1999). Signaling through focal adhesion kinase. Prog. Biophys. Mol. Biol.

[21] De Mali K.A., Wennerberg K., Burridge K. (2003). Integrin signaling to the actin cytoskeleton. Curr. Opin. Cell. Biol.

[22] Das K., Bose S., Bandyopadhyay A. (2007). Surface modification and cell-materials interactions with anodized Ti. Acta Biomater.

[23] Yamamura M., Modlin R.L., Ohmen J.D., Moy R.L. (1993). Local expression of antiinflammatory cytokines in cancer. J. Clin. Invest.

[24] Prehn R.T. (1993). Tumor immunogenicity: How far can it be pushed. Proc. Natl. Acad. Sci. USA.

[25] Gehan E.A., Walker W.D. (1977). Prognostic factors for patients with brain tumors. Natl. Cancer Inst. Monogr.

